# Activation of Phosphatidylcholine-Specific Phospholipase C in Breast and Ovarian Cancer: Impact on MRS-Detected Choline Metabolic Profile and Perspectives for Targeted Therapy

**DOI:** 10.3389/fonc.2016.00171

**Published:** 2016-08-02

**Authors:** Franca Podo, Luisa Paris, Serena Cecchetti, Francesca Spadaro, Laura Abalsamo, Carlo Ramoni, Alessandro Ricci, Maria Elena Pisanu, Francesco Sardanelli, Rossella Canese, Egidio Iorio

**Affiliations:** ^1^Molecular and Cellular Imaging Unit, Department of Cell Biology and Neurosciences, Istituto Superiore di Sanità, Rome, Italy; ^2^Department of Biomedical Sciences for Health, Università degli Studi di Milano, Research Hospital Policlinico San Donato, Milan, Italy

**Keywords:** choline metabolism, phosphatidylcholine phospholipase C, choline kinase, breast cancer, ovarian cancer, magnetic resonance spectroscopy, targeted therapy

## Abstract

Elucidation of molecular mechanisms underlying the aberrant phosphatidylcholine cycle in cancer cells plays in favor of the use of metabolic imaging in oncology and opens the way for designing new targeted therapies. The anomalous choline metabolic profile detected in cancer by magnetic resonance spectroscopy and spectroscopic imaging provides molecular signatures of tumor progression and response to therapy. The increased level of intracellular phosphocholine (PCho) typically detected in cancer cells is mainly attributed to upregulation of choline kinase, responsible for choline phosphorylation in the biosynthetic Kennedy pathway, but can also be partly produced by activation of phosphatidylcholine-specific phospholipase C (PC-PLC). This hydrolytic enzyme, known for implications in bacterial infection and in plant survival to hostile environmental conditions, is reported to be activated in mitogen- and oncogene-induced phosphatidylcholine cycles in mammalian cells, with effects on cell signaling, cell cycle regulation, and cell proliferation. Recent investigations showed that PC-PLC activation could account for 20–50% of the intracellular PCho production in ovarian and breast cancer cells of different subtypes. Enzyme activation was associated with PC-PLC protein overexpression and subcellular redistribution in these cancer cells compared with non-tumoral counterparts. Moreover, PC-PLC coimmunoprecipitated with the human epidermal growth factor receptor-2 (HER2) and EGFR in HER2-overexpressing breast and ovarian cancer cells, while pharmacological PC-PLC inhibition resulted into long-lasting HER2 downregulation, retarded receptor re-expression on plasma membrane and antiproliferative effects. This body of evidence points to PC-PLC as a potential target for newly designed therapies, whose effects can be preclinically and clinically monitored by metabolic imaging methods.

## Phosphatidylcholine-Specific Phospholipase C in Living Systems

Phosphatidylcholine-specific phospholipase C (EC 3.1.4.3, here abbreviated as PC-PLC) is responsible for hydrolysis of this glycerophospholipid into phosphocholine (PCho) and 1,2-diacylglycerols (DAG).

Phospholipases of this class are known to be important secreted pathogenicity factors in some bacteria, parasites, and fungi (1, 2) in which they act as lytic agents against eukaryotic cells and interfere with the host immune defense. Some bacterial PC-PLCs are also involved in lipid remodeling in response to phosphate-limiting conditions. Amino acid sequences and encoding genes have been identified for various toxic and non-toxic PC-PLCs produced by Gram-positive and Gram-negative bacteria. Since some PC-PLCs play important roles in the pathogenesis of diseases, they could also form components of vaccines.

Phospholipases C endowed with a broader substrate specificity (collectively called NPC, as an acronym for “non-specific phospholipases C”) were discovered, sequenced, and cloned in plants as a novel family of phospholipid-cleaving enzymes homologous to bacterial PC-PLCs and responsible for lipid conversion under phosphate-limiting conditions (3). Notably, phosphatidylcholine-hydrolyzing members of the NPC family in *Arabidopsis* were implicated in stress response to phytohormones, root development, and tolerance to adverse environmental conditions (3).

Phosphatidylcholine-specific phospholipase C activity is reported to be an essential source of phospholipid-derived signaling in animal cells (4, 5) in which this phospholipase can be implicated in various intracellular regulatory mechanisms, including long-term cell response to mitogens (6–9); cell cycle regulation and cell proliferation (8, 10, 11); programmed cell death (12, 13); activation of cells of the immune system (14–22); cell transformation (23, 24); oncogene-driven cell signaling and tumor progression (25–28); and cell differentiation of tumoral and non-tumoral cells (29–34).

Phosphatidylcholine-specific phospholipase C isoforms of varying molecular weights have been isolated from mammalian sources (35–37). However, differently from phosphatidylinositol-bis-phosphate specific PLCs (PIP_2_-PLCs), well-recognized key regulatory enzymes of cell growth, development, and stress responses in living organisms, a slower progress has been so far achieved in the molecular characterization of PC-PLCs in animal cells, in which these phospholipases have not yet been sequenced and cloned. For these reasons, the role of PC-PLCs in mammalian cells has remained elusive until recently. Despite these limitations, the PC-PLC protein expression could be effectively investigated in mammalian cells using cross-reacting polyclonal antibodies raised in rabbits against bacterial PC-PLCs, as first described by Clark et al. (37). Using these antibodies, a 66-kDa PC-PLC isoform has been detected in various mammalian cell systems, such as mouse NIH-3T3 fibroblasts (8, 38), synaptic endings (39, 40), epithelial ovarian cancer (EOC) cells and surgical specimens (26, 27), breast cancer (BC) (28) and hepatoma cells (11, 30, 41). Furthermore, near-infrared probes capable to non-invasively detect PC-PLC in experimental animals have been developed and their utility tested for *in vivo* cancer imaging (42).

An increasing interest in filling the existing gaps in the molecular and genomic characterization of mammalian PC-PLCs arises from accruing evidence that protein overexpression, subcellular redistribution, and activation of this enzyme in tumor cells represent relevant features of the aberrant choline phospholipid metabolism in cancer (43). In addition, evidence for a physical interaction of PC-PLC with the human epidermal growth factor receptor-2 (HER2) and EGFR is provided by coimmunoprecipitation tests on HER2-overexpressing BC (28) and EOC cells.^1^ Pharmacological PC-PLC inhibition is associated in these cells with long-lasting HER2 downmodulation and induction of antiproliferative effects, suggesting a role for PC-PLC activity in controlling HER2-driven tumorigenicity. Furthermore, inhibition of PC-PLC is associated with loss of mesenchymal traits in the highly metastatic triple-negative MDA-MB-231 cells and with decreased *in vitro* cell migration and invasion capabilities, suggesting a pivotal role for PC-PLC in BC cell differentiation (34).

This article provides a brief overview on metabolic and functional features of PC-PLC in BC and EOC cells and outlines some perspectives offered by further elucidation of the impact of this phospholipase on cancer cell biology and therapy targeting.

## PC-PLC in Breast and Ovarian Cancer Cells

### PC-PLC Activation and Contribution to Elevated Phosphocholine Production

**Figure 1:**
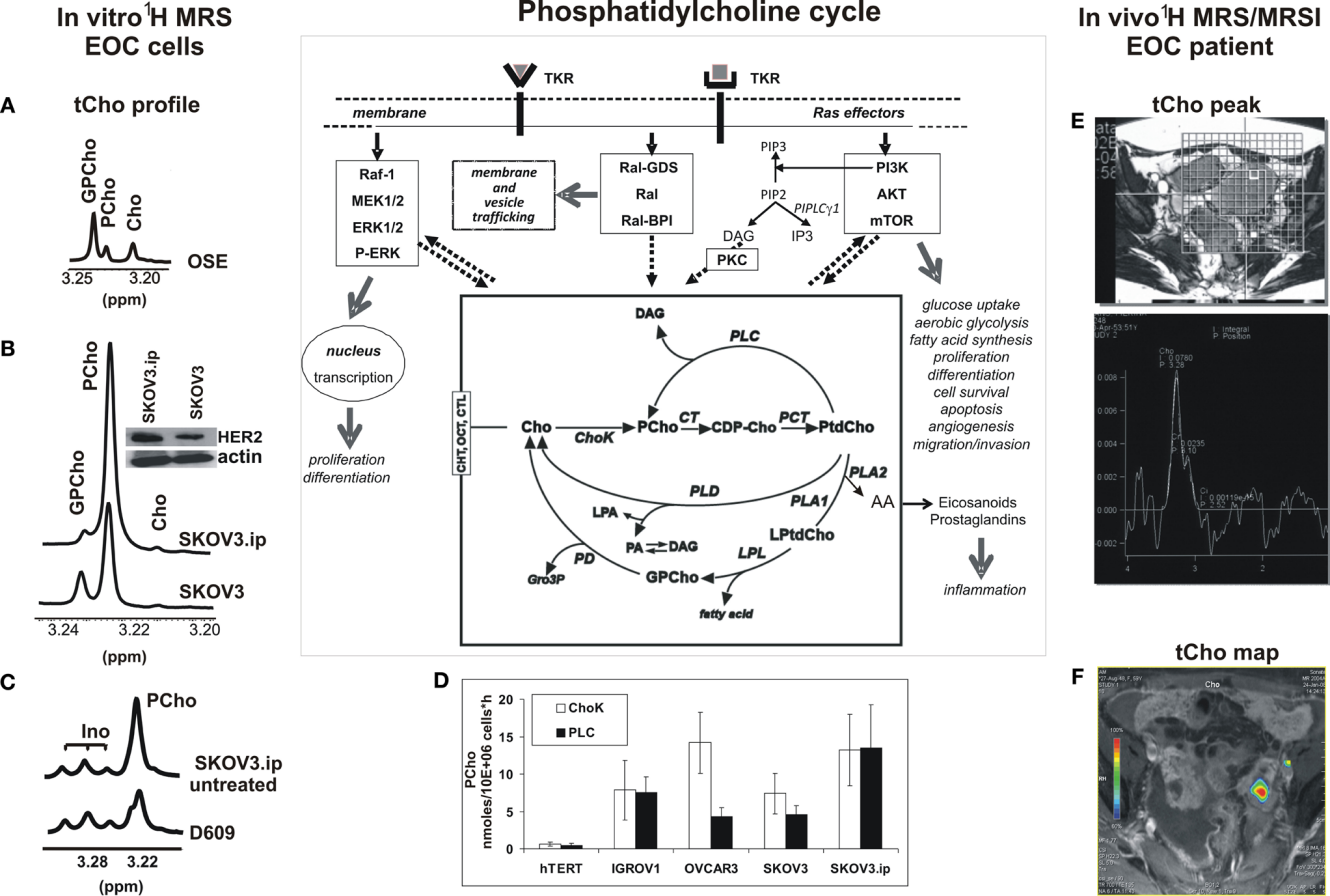
**Detection of the ^1^H-MRS total choline (tCho) metabolic profile in epithelial ovarian cancer (EOC) cells and in MRS/MRSI clinical examinations**. The phosphatidylcholine cycle and its links with tyrosine kinase receptors’ stimulation, post-receptor signaling pathways, and cancer cell biological features are sketched in the central scheme. **(A–C)** High resolution 9.4-T ^1^H-MRS tCho profile of aqueous extracts of **(A)** ovarian surface epithelial cells (OSE); **(B)** SKOV3 cell line and its *in vivo*-passaged cell variant SKOV3.ip, characterized by higher *in vivo* tumorigenicity and twofold higher HER2 protein expression level (in the insert, Western blot analysis of HER2, 185 kDa); **(C)** SKOV3.ip cells exposed for 24 h to the competitive PC-PLC inhibitor D609, compared with control untreated cells. **(D)** Absolute activity rates (nmol/10^6^ cells × h, mean ± SD) of ChoK and PC-PLC measured in aqueous extracts of different EOC cell lines, compared with those of non-tumoral immortalized hTERT cells (number of independent experiments, *n* ≥ 3 for all cell lines). **(E,F)** Examples of *in vivo* detection of **(E)** ^1^H-MRS tCho peak (3.2 ppm) in a single voxel and **(F)** tCho map obtained by MRSI in EOC patient examined at 1.5 T. For further details, see Ref. (25, 27, 49–51). The central scheme was adapted from Figure 1 of Ref. (43). Abbreviations: *Enzymes*: ChoK, choline kinase (EC 2.7.1.32); CT, cytidylyltransferase (EC 2.7.7.15); LPL, lysophospholipase (EC 3.1.1.5); PCT, phosphocholine transferase (EC 2.7.8.2); PD, glycerophosphocholine phosphodiesterase (EC 3.1.4.2); PLA1, phospholipase A1 (EC 3.1.1.32); PLA2, phospholipase A2 (EC 3.1.1.4); PLC, phosphatidylcholine-specific phospholipase C (EC 3.1.4.3); PLD, phospholipase D (EC 3.1.4.4). *Metabolites*: AA, arachidonic acid; CDP-Cho, cytidine diphosphate-choline; Cho, free choline; DAG, diacylglycerol; GroP, *sn*-glycerol-3-phosphate; GPCho, glycerophosphocholine; Ino, myo-inositol; LPA, lysophosphatidate; LPtdCho, lysophosphatidylcholine; PA, phosphatidate; PCho, phosphocholine; PtdCho, phosphatidylcholine; tCho, total choline-containing metabolites (GPCho + PCho + Cho). *Transporters*: CHT, choline high-affinity transporters; CTL, choline transporter-like proteins; OCT, organic cation transporters.

Phosphatidylcholine, the most abundant phospholipid of eukaryotic cells, plays the double role of basic structural component of cell membranes and precursor of agonist-induced signaling lipids (44) through a network of enzymatic reactions known as the phosphatidylcholine cycle (45) (scheme in Figure 1). The agonist-induced production and utilization within this cycle of signaling lipids, such as DAG, phosphatidate, lysophosphatidylcholine, and arachidonic acid, are associated with changes in the fluxes and steady-state levels of water-soluble phosphatidylcholine metabolites, such as PCho, glycerophosphocholine (GPCho), and free choline (Cho), main components of the so-called total choline (tCho) metabolic profile.

Magnetic resonance spectroscopy (MRS) and spectroscopic imaging (MRSI) represent powerful means to detect changes induced in the tCho pool by oncogene-driven activation of phosphatidylcholine cycle enzymes in cancer and to evaluate the response of the tCho profile to agents targeted against selected enzymes (43, 46–48). Figure 1 shows examples of (i) the ^1^H-MRS tCho profile in aqueous extracts of normal ovarian surface epithelial cells (OSE, Figure 1A) compared with the EOC cell line SKOV3 and its *in vivo*-passaged cell variant SKOV3.ip, characterized by higher HER2 overexpression (Figure 1B) and enhanced *in vivo* tumorigenicity (25, 27, 49, 50); (ii) the effects induced on the SKOV3.ip tCho profile by cell exposure to D609, a competitive PC-PLC inhibitor (Figure 1C); (iii) the simultaneous activation of ChoK and PC-PLC in different EOC cell lines versus hTERT, a non-tumoral immortalized ovarian cell line (Figure 1D); and (iv) examples of *in vivo* detection of the ^1^H-MRS tCho peak (3.2 ppm) in a selected voxel (Figure 1E) and a tCho map obtained by 3D-MRSI in a EOC patient (Figure 1F) (51).

The ^1^H-MRS PCho signal increased 3.6- to 6.5-fold in the investigated EOC cell lines compared with non-tumoral counterparts (25, 27, 50). Notably, the 2-fold higher HER2 expression of SKOV3.ip versus SKOV3 cells was associated with a 1.7-fold higher PCho content (50). An elevated PCho level was the principal cause of the higher tCho pool detected in these cancer cells, in agreement with the general view that an increased tCho represents a metabolic signature of malignancy (43, 46–48, 52, 53). The enhanced PCho production in cancer cells is currently attributed to the upregulation of choline kinase (43, 46), the enzyme committed to choline phosphorylation in the biosynthetic Kennedy pathway. This enzyme (notably the ChoK-alpha isoform) has been proposed as a new target for cancer therapy (54, 55). A substantial contribution to the production of the intracellular PCho pool in cancer cells may, however, also derive from PC-PLC activation (see scheme in Figure 1). The question therefore arises on the relative contributions possibly given by PC-PLC activation to PCho production in different EOC cells. Measurements in our laboratory (Figure 1D) showed that a mean 12- to 24-fold activation of ChoK was paralleled by a 10- to 30-fold activation of PC-PLC in four EOC cell lines (IGROV1, OVCAR3, SKOV3, and SKOV3.ip) versus the non-tumoral hTERT cell line (in which both enzymes had a basal activity of about 0.5 nmol/10^6^ cells × h). In some EOC cell lines (IGROV1, SKOV3.ip), ChoK and PC-PLC showed very similar activity rates, suggesting about equal contributions of the two enzymes to the PCho production. Accordingly, there was a 40–50% decrease of the PCho signal in SKOV3.ip cells following 24-h exposure to the competitive PC-PLC inhibitor D609 (example in Figure 1C). In another cell line (OVCAR3), the mean activity rate of ChoK was instead fourfold higher than that of PC-PLC. Overall, these data showed that the contribution of PC-PLC to the PCho production could vary, according to the cell line, between 20% and 50%, in substantial agreement with the effects of D609 or si-RNA ChoK silencing on the PCho levels measured in different EOC cells [Figure 1C; (27, 56)].

This body of evidence supports the conclusion that PC-PLC can contribute to a substantial extent to the accumulation of PCho in EOC cells. This evidence may allow a better interpretation of changes occurring in the *in vivo* MRS- and MRSI-detected tCho peak in preclinical EOC models (57) and in EOC clinical lesions (examples in Figures 1E,F) during tumor progression, response to treatment, or relapse.

**Figure 2:**
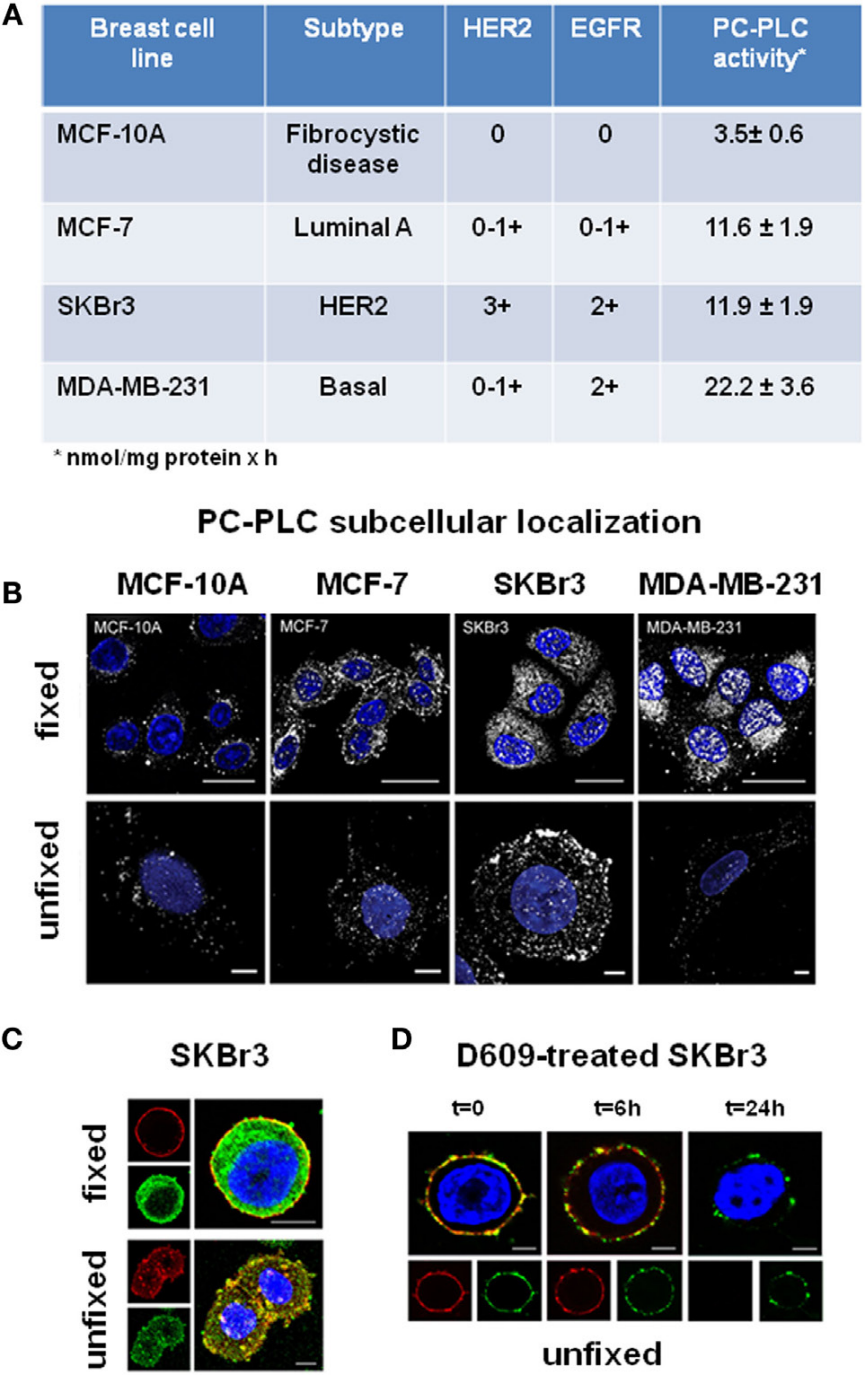
**Subcellular localization of PC-PLC in human BC cell lines**. **(A)** PC-PLC activity rates (nmol/mg protein × h, mean ± SD, *n* ≥ 3) measured in MCF-7, SKBr3, and MDA-MB-231 BC cell lines, possessing different levels of HER2 and EGFR expression, compared with that of the human mammary epithelial cell line of fibrocystic origin MCF-10. **(B)** Confocal laser scanning microscopy (CLSM) examinations (3D reconstruction images) of the same cell lines as in **(A)**. Cells were either fixed and permeabilized (upper panels, scale bars 20 μm) or maintained unfixed (bottom panels, scale bars 5 μm) and stained for PC-PLC detection with rabbit anti-PC-PLC antibodies (pseudo-color gray); nuclei were stained with DAPI (blue). **(C)** Colocalization of PC-PLC and HER2 on plasma membrane of SKBr3 cells. CLSM detection of PC-PLC and HER2 was performed on fixed and permeabilized (upper panels) or unfixed cells (bottom panels, 3D reconstruction images) using rabbit polyclonal anti-PC-PLC antibodies (green) and anti-HER2 W6/100 monoclonal antibody (red). Colocalization areas are represented in yellow. Scale bars, 8 μm. **(D)** HER2 downmodulation in SKBr3 cells exposed for different time intervals to the competitive PC-PLC inhibitor D609. CLSM analyses of unfixed SKBr3 cells cultured in complete medium in absence (*t* = 0) or presence of D609 (6 and 24 h). After washing, cells were stained with anti-PC-PLC (green) and anti-HER2 antibodies (red). For further details, see Ref. (28, 34).

Regarding human BC cells, a significant 2- to 5.5-fold ChoK activation was detected by Eliyahu and colleagues in cell lines of different subtypes (58), such as luminal-A MCF-7, HER2-positive SKBr3, and basal-like EGFR-positive MDA-MB-231 (respective activity rates 21 ± 3, 43 ± 6, and 17 ± 2 nmol/mg protein × h), versus mammary epithelial cells (8.0 ± 2.0 nmol/mg protein × h). Enzymatic assays in our laboratory (34) showed threefold to sixfold higher PC-PLC activity rates (ranging between 12 ± 2 and 22 ± 4 nmol/mg protein × h) in these BC cells versus the fibrocystic MCF-10 cell line (Figure 2A). Overall, these data suggest that the contribution of PC-PLC to the intracellular PCho production also varied in these BC cells between 20% and 50%, as reported above for EOC cell lines.

### PC-PLC Overexpression and Subcellular Redistribution

Western blot analyses showed that the reported threefold to sixfold activation of PC-PLC in BC cell lines was associated with a threefold to sixfold elevated PC-PLC protein expression (34), the highest value being detected in the triple-negative MDA-MB-231 cells. Confocal laser scanning microscopy (CLSM) of fixed and permeabilized cancer cells (Figure 2B, top panels) showed that the enzyme was localized both in nuclear and cytoplasmic compartments. Only a few PC-PLC-positive granules, mainly localized in perinuclear areas and absent in the nuclear matrix, were instead detected in the non-tumoral MCF-10A cells. CLSM of unfixed cells showed that PC-PLC massively accumulated on plasma membrane of the HER2-positive SKBr3 cells, but not on that of cells with basal HER2 expression (Figure 2B, bottom panels). PC-PLC extensively colocalized with HER2 in raft domains of plasma membrane of SKBr3 cells [Figure 2C; (28)], in which coimmunoprecipitation tests showed a physical association of PC-PLC with HER2 and with other members of the ErbB family, such as EGFR and HER3.

Regarding PC-PLC distribution in EOC cells, a massive accumulation of this phospholipase was detected by CLSM on plasma membrane of all investigated cell lines and on that of cancer cells isolated from patient peritoneal exudate, but not on OSE cells (26). The enzyme extensively colocalized with β1-integrin in non-raft domains of EOC cells (26). The extent of colocalization of the two proteins substantially decreased on membrane of serum-deprived cancer cells, but returned to the original level upon cell restimulation by platelet-derived growth factor. These data warrant further investigations on the functional role of the interaction between PC-PLC and β1-integrin, an adhesion protein well-known for its involvement in metastatic spread (59). Furthermore, CLSM and coimmunoprecipitation experiments showed an extensive colocalization and physical association of PC-PLC with HER2 on plasma membrane of SKOV3 and SKOV3.ip cells, confirming the interest of further investigating the role of this phospholipase in regulating HER2 overexpression in ovarian cancer.

### Effects of PC-PLC Inhibition

Exposure of BC cell lines to D609 led to cell proliferation arrest, changes in cell morphology, and formation of lipid bodies typical of BC cell differentiation (34). Moreover, in the poorly differentiated MDA-MB-231 cells, PC-PLC inhibition was associated with progressive decreases of mesenchymal traits, such as vimentin and N-cadherin expression, reduced galectin-3 and milk fat globule EGF-factor 8 levels, β-casein formation, and decrease of *in vitro* cell migration and invasion (34). We therefore proposed that the inhibition of this phospholipase can be envisaged as a means to promote differentiation of metastatic BC cells, with potential therapeutic effects.

Besides inducing cell differentiation, exposure of SKBr3 cells to D609 resulted in a progressive downmodulation of both HER2 and PC-PLC on plasma membrane, an effect already evident at 6 h and almost complete at 24 h (Figure 2D). This effect was associated with HER2 internalization and lysosomal degradation, long-lasting retardation of re-expression of this receptor on membrane, and an about 50% decrease in the overall protein expression (28). Notably, no substantial influence on HER2 externalization is known to be exerted in these cells using Trastuzumab (60), the anti-HER2 humanized monoclonal antibody mostly used at the clinical level, whose *in vivo* therapeutic efficacy is mainly attributed to antibody-mediated cytotoxicity. This body of evidence suggests that PC-PLC inhibition may be envisaged as a potential alternative approach to counteract the tumorigenic effects of HER2, especially in cases of resistance (or contraindication) to current anti-HER2 treatment.

Long-standing decreases of HER2 and phospho-HER2 contents, reduced HER2 mRNA expression, and cell cycle arrest were also detected in the highly tumorigenic SKOV3.ip EOC cells exposed to a non-apoptotic dose of D609^1^. Furthermore, reduced tumor growth and decrease in HER2 and Ki67 immunostaining were detected in SKOV3.ip xenografts upon *in vivo* treatment with D609, pointing to the interest of further evaluating the potential role of PC-PLC as a therapy target in preclinical HER2-overexpressing EOC models.

## Future Research Directions

In summary, PC-PLC is overexpressed and activated in BC and EOC cells, and pharmacological PC-PLC inhibition can lead to downregulation of HER2 and induction of antiproliferative effects. Furthermore, cell differentiation and decreases in cell migration and invasion were induced in the highly metastatic MDA-MB-231 BC cell line exposed to the PC-PLC inhibitor. These findings point to PC-PLC as a potential target (or cotarget) for anticancer therapy, especially in cases of resistance or contraindications to currently adopted treatments. In this context, the following issues appear worth of further elucidation:

### Molecular Mechanisms Responsible for Oncogene-Driven PC-PLC Activation and Impact on Molecular Imaging

The 1.7-fold increase in PC-PLC activity in a SKOV3 cell variant (SKOV3.ip) endowed with a 2-fold higher HER2 overexpression (50) and the over 3-fold increase in PC-PLC activity in the HER2-positive SKBr3 cells (34) warrant further investigations on the molecular mechanisms linking PC-PLC activation with HER2 overexpression and oncogenic effects of HER2 and HER2–EGFR heterodimers. Interestingly, the PC-PLC inhibitor D609, but not Trastuzumab, induced decreases of HER2 expression and cell proliferation in the Trastuzumab-resistant SKBr3 cell line. Conversely D609, either alone or in combination with Trastuzumab, induced in the Trastuzumab-sensitive BT-474 cells a decrease in cell proliferation comparable to that induced using Trastuzumab alone (28).

The sixfold activation of PC-PLC in MDA-MB-231 BC cells and its strong nuclear localization (Figures 2A,B) warrant investigations on the relationships between PC-PLC and EGFR overexpression in triple-negative BC cells and their metastatic derivatives.

Overexpression of EGFR and c-Src in BC cells has been reported to synergistically increase ChoK-alpha protein expression and activity levels (61). Further elucidation of EGFR-driven mechanisms responsible for the activation of both ChoK-alpha or PC-PLC in BC cells may lead to novel multi-targeted anticancer therapies, whose effects could be preclinically and clinically monitored by MRS-based and optical metabolic imaging methods.

Integration of MRS with ^11^C or ^18^F choline-based positron emission tomography (PET) may allow discrimination of the contributions to PCho production, provided by the biosynthetic and catabolic pathways, respectively. In fact, while both ChoK and PC-PLC can contribute to the MRS-detected PCho signal, the choline-PET standardized uptake value (SUV) mainly reflects choline transport and phosphorylation in the Kennedy pathway, with negligible contributions from products of radiolabeled phosphatidylcholine catabolism in a time window of about 1 h (the duration of a typical choline-PET examination) (43).

### Molecular and Functional Characterization of the Interaction between PC-PLC and Receptors of the ErbB Family

The interaction of an activated PC-PLC isoform with HER2, detected by coimmunoprecipitation tests, appears essential to the functional localization of this receptor in BC cells, since PC-PLC inhibition induces a long-lasting HER2 downmodulation (28). The association of these effects with reduced cell proliferation, induction of cell differentiation, and decrease in cell migration and invasion suggests that an activated PC-PLC may act as a sort of chaperone for HER2. A local overproduction of DAG by enhanced PC-PLC-mediated phosphatidylcholine hydrolysis could mediate this action. A local accumulation of DAG can, in fact, perturb the phospholipid bilayer, alter protein-lipid interactions, and influence the formation of microdomains, thus modifying the exposure of surface membrane receptors and affecting their recycling between membrane and inner cell compartments (62, 63). Interestingly, ChoK-alpha has also been proposed to act as a chaperone for the androgen receptor, a ligand-inducible transcription factor of the nuclear hormone receptor superfamily, critically involved in prostate cancer progression (64). The newly suggested role of ChoK and PC-PLC as regulators of expression for oncogenes and growth factor receptors may lead to new targeted or multi-targeted anticancer therapies.

### Structural and Genomic Characterization of PC-PLC Isoforms Active in Cancer Cells

This body of evidence points to the need for overcoming the current limitations deriving from (a) the scarce attention so far devoted to the genomic and structural characterization of mammalian PC-PLC isoforms and (b) the availability until now of only one PC-PLC inhibitor, D609, a synthetic tricyclic compound having a xanthate group, possessing antiviral, anti-tumor, anti-inflammatory, and anti-apoptosis properties and used as a lipid-related enzyme inhibitor for the past three decades (65).

Steps forward in translational oncology may be expected from:

(a) investigating the role of PC-PLC expression and the effects of PC-PLC inhibition in BC and EOC stem cells, to identify potential ways to reinforce therapeutic strategies aimed to eradicate malignancies (66, 67);
(b) exploring in preclinical models the correlation between PC-PLC activity and disease progression or response to therapy, as a ground for evaluating the impact of PC-PLC activation on clinical outcome.

## Conclusion

Recent evidence shows protein overexpression and enzymatic activation of PC-PLC in BC and EOC cells compared with non-tumoral counterparts.

The activation of PC-PLC can contribute to 20–50% of the production of intracellular PCho in BC and EOC cells.

Phosphatidylcholine-specific phospholipase C activation is associated in these cancer cells with its protein overexpression and subcellular redistribution, the underlying molecular mechanisms deserving further elucidation.

Phosphatidylcholine-specific phospholipase C interacts with HER2 and EGFR in HER2-overexpressing BC and EOC cells, while its pharmacologic inhibition may play a pivotal role in HER2 downmodulation, reduction of cell proliferation, and cancer cell differentiation.

Overall, this body of evidence supports the interest of evaluating the possible role of this phospholipase as a key metabolic target for anticancer therapy.

## Ethics Statement

The study on patients mentioned in this Perspective article (Figures 1E,F) was approved by the Institutional Review Board of the Research Hospital Policlinico San Donato Milanese, Italy. Written informed consent was obtained from all patients. Between July 2007 and April 2009, the study prospectively enrolled a series of patients with already known ovarian masses, who were consecutively referred to the Radiology Unit of Policlinico San Donato Milanese for presurgical staging. Exclusion criteria were the lack of informed consent and general contraindications to MRI or to the intravenous administration of contrast material. All patients underwent surgery with removal of the mass and histopathological examination. Further details are given in Ref. (51).

## Author Contributions

The manuscript was written by FP; revised by EI, SC, FSp, RC, FSa, and FP; read and approved by all coauthors.

## Conflict of Interest Statement

The authors declare that the research was conducted in the absence of any commercial or financial relationships that could be construed as a potential conflict of interest.

## Footnotes

^1^Paris L, Podo F, Spadaro F, Abalsamo L, Pisanu ME, Ricci A, et al. Phosphatidylcholine-specific phospholipase C inhibition reduces HER2-overexpression and tumor growth in a highly tumorigenic ovarian cancer model. Manuscript in preparation (2016).
